# Alternative splicing and gene expression play contrasting roles in the parallel phenotypic evolution of a salmonid fish

**DOI:** 10.1111/mec.15817

**Published:** 2021-02-18

**Authors:** Arne Jacobs, Kathryn R. Elmer

**Affiliations:** ^1^ Institute of Biodiversity, Animal Health and Comparative Medicine, College of Medical, Veterinary & Life Sciences University of Glasgow Glasgow UK; ^2^ Department of Natural Resources Cornell University Ithaca NY USA

**Keywords:** adaptive divergence, alternative splicing, convergent evolution, ecological speciation, gene expression, parallel evolution, salmonid

## Abstract

Understanding the contribution of different molecular processes to evolution and development is crucial for identifying the mechanisms of adaptation. Here, we used RNA‐sequencing data to test the importance of alternative splicing and differential gene expression in a case of parallel adaptive evolution, the replicated postglacial divergence of the salmonid fish Arctic charr (*Salvelinus alpinus*) into sympatric benthic and pelagic ecotypes across multiple independent lakes. We found that genes differentially spliced between ecotypes were mostly not differentially expressed (<6% overlap) and were involved in different biological processes. Differentially spliced genes were primarily enriched for muscle development and functioning, while differentially expressed genes were involved in metabolism, immunity and growth. Furthermore, alternative splicing and gene expression were mostly controlled by independent *cis*‐regulatory quantitative trait loci (<3.4% overlap). *Cis*‐regulatory regions were associated with the parallel divergence in splicing (16.5% of intron clusters) and expression (6.7%–10.1% of differentially expressed genes), indicating shared regulatory variation across ecotype pairs. Contrary to theoretical expectation, we found that differentially spliced genes tended to be highly central in regulatory networks (“hub genes”) and were annotated to significantly more gene ontology terms compared to nondifferentially spliced genes, consistent with a higher level of pleiotropy. Together, our results suggest that the concerted regulation of alternative splicing and differential gene expression through different regulatory regions leads to the divergence of complementary processes important for local adaptation. This provides novel insights into the importance of contrasting but putatively complementary molecular processes in rapid parallel adaptive evolution.

## Abstract 2

see also the Perspective by Sarah J. Salisbury, M. Lisette Delgado and Anne C. Dalziel.

## 1 INTRODUCTION

Since Turesson first used the term “ecotype” nearly a century ago to describe genetically distinct populations that are adapted to alternative ecological environments (Turesson, 1922), we have gained substantial insights into the genetics of ecological adaptation. Yet our knowledge of the molecular and regulatory mechanisms linking environmental influences, functional variation, and the development and evolution of new phenotypes in nature is still limited (Lewis & Reed, 2019; Verta & Jones, 2019). Divergence in gene expression has been strongly implicated in the rapid evolution and development of adaptive and divergent phenotypes, particularly as *cis*‐regulatory mutations are thought to exhibit fewer deleterious effects than protein‐coding changes (Alvarez et al., 2015; Campbell‐Staton et al., 2017; Filteau et al., 2013; Jacobs et al., 2019; Mack et al., 2018; Manousaki et al., 2013; McGirr & Martin, 2018; Prud’homme et al., 2007; Verta & Jones, 2019). However, post‐transcriptional processes, which have been suggested to play a substantial role in generating phenotypic variation (Bush et al., 2017; Howes et al., 2017; Li et al., 2016; Parenteau et al., 2019; Singh et al., 2017), have rarely been evaluated in cases of rapid adaptation in natural systems (Howes et al., 2017; Mallarino et al., 2017; Singh et al., 2017; Wang et al., 2018). This raises an important knowledge gap regarding the contribution and interaction of different molecular mechanisms in the evolution of ecologically adaptive phenotypes.

The alternative splicing of pre‐mRNA transcripts is a post‐transcriptional process that has been associated with phenotypic diversification in eukaryotes (Bush et al., 2017). Alternative splicing leads to the formation of distinct transcripts (“isoforms”) through the retention or removal of different exons and introns from the immature mRNA of a single gene (Bush et al., 2017; Nilsen & Graveley, 2010; Stamm et al., 2005). These isoforms can either encode structurally distinct proteins and result in the functional diversification of the proteome (Bush et al., 2017; Nilsen & Graveley, 2010), or alter the regulation of transcript abundance through the formation of nonsense transcripts, which will be efficiently removed (Aznarez et al., 2018; Grantham & Brisson, 2018). As splicing enables the extension and regulation of the transcriptome without requiring changes to expression levels or alteration of the ancestral isoform function, it is potentially less constrained than expression variation. In eukaryotes, the majority of genes undergo splicing at some point during development (Grau‐Bové et al., 2018). Splicing occurs through a dynamic ribonucleoprotein complex, the spliceosome (Lee & Rio, 2015). Studies in model organisms have shown that the expression and splicing of genes are mostly controlled by different genetic loci, indicating these processes can evolve independently (Li et al., 2016).

Patterns of alternative splicing have been found to differ between closely related species (Harr & Turner, 2010; Singh et al., 2017) and the differential splicing of individual large‐effect genes has in some cases been linked to the rapid evolution of ecologically relevant phenotypic traits (Howes et al., 2017; Mallarino et al., 2017; Verta et al., 2020). Similar to gene expression, alternative splicing is regulated through genetic *cis*‐ and *trans*‐regulatory differences, although splicing and expression seem to be largely regulated by different regulatory elements (Gao et al., 2015; Lee & Rio, 2015; Li et al., 2016; Smith et al., 2018; Verta et al., 2020; Wang et al., 2018), facilitating different evolutionary responses. However, alternative splicing is also sensitive to environmental differences and has been linked for example to pea aphid polyphenisms (Grantham & Brisson, 2018) and queen pheromone exposure in ants and bees (Holman et al., 2019). However, compared to the regulation of gene expression divergence under different evolutionary scenarios (e.g. Mack et al., 2018; Mack & Nachman, 2017; Signor & Nuzhdin, 2018; Verta & Jones, 2019), the regulation and evolution of alternative splicing on a genome‐wide scale, and particularly its contributions to local adaptation and rapid phenotypic evolution, are less well understood (Howes et al., 2017; Smith et al., 2020). In principle, alternative splicing and gene expression can evolve independently, either playing contrasting roles by affecting different genes and biological pathways (Grantham & Brisson, 2018), or playing redundant roles by affecting the same genes (Healy & Schulte, 2019; Singh et al., 2017). Due to putatively weaker functional constraints, alternative splicing might be able to alter the expression or isoform diversity of more strongly constrained genes, such as highly pleiotropic genes, while retaining essential expression levels or isoforms (McGirr & Martin, 2018; Papakostas et al., 2014). It has been further suggested that splicing diverges faster between vertebrate species than gene expression, which could potentially facilitate rapid adaptation, yet to date most comparative studies have focused on timescales over millions of years (Barbosa‐Morais et al., 2012; Merkin et al., 2012). Thus, despite our growing knowledge of the importance of alternative splicing in phenotypic evolution, the role of differential splicing has rarely been studied for cases of rapid ecological adaptation in natural systems on postglacial timescales and on genome‐wide scales, restricting our understanding of its influence and consistency in adaptive phenotypic diversification.

To address this gap, we assess the pattern of alternative splicing associated with rapid phenotypic divergence in a natural and replicated system of ecological speciation. Arctic charr (*Salvelinus alpinus*) is a salmonid fish with a holarctic distribution and numerous and well‐studied cases of independent parallel divergence into ecotypes, which differ in a range of ecological and phenotypic aspects along the depth axis within freshwater lakes (Elmer, 2016; Jonsson & Jonsson, 2001). Benthic ecotypes mostly occupy the profundal or littoral habitat, show lower swimming activity, and usually have larger eyes and deeper and more robust heads, allowing them to better handle benthic prey (Adams & Huntingford, 2002a, 2002b; Alekseyev et al., 2002; Jonsson & Jonsson, 2001; Klemetsen, 2002). In contrast, pelagic ecotypes occupy the open water where they forage for plankton and are thus more active swimmers, with mostly smaller and more slender heads (Adams & Huntingford, 2002a, 2002b; Alekseyev et al., 2002; Jonsson & Jonsson, 2001; Klemetsen, 2002). These divergent ecotypes have evolved independently following the last glacial maximum 10,000–15,000 years ago (Jacobs et al., 2020). Patterns of genomic differentiation and evolutionary histories differ widely across Arctic charr ecotype pairs, while patterns of gene expression divergence are more predictable and consistent across populations (Guðbrandsson et al., 2018; Jacobs et al., 2020). Both heritable genetic and plastic changes have been shown to contribute to this ecotype divergence (Adams & Huntingford, 2002a,2002b,^2004^; Garduno‐Paz et al., 2012; Jacobs et al., 2020; Klemetsen, 2002).

**FIGURE 1:**
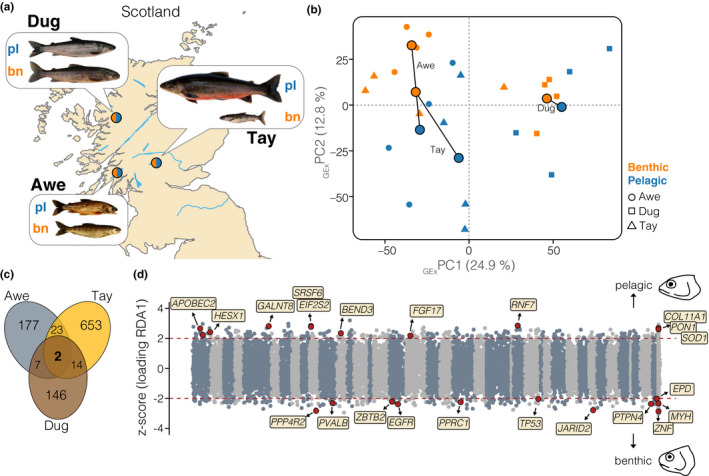
Sampling and differential gene expression. (a) Map showing the locations of the focal populations (Awe, Tay and Dughaill [Dug]). Representative pictures of the benthic and pelagic ecotypes per lake are shown. Coordinates and additional sample information are given in Table 1. (b) PCA based on expression data (GEx‐PCA) of all genes (*n* = 19,623) for all individuals (*n* = 24). Round large points show the respective centroids for each ecotype, and sympatric ecotypes are connected by vectors. Small unicolour points represent individuals. Populations are coded by shape and ecotypes by colour. (c) Venn diagram displaying the amount of overlap of differentially expressed genes between sympatric ecotypes (FDR < 0.05) across lakes. (d) Distribution of z‐scores derived from the RDA, depicting association of gene expression with ecotype across the genome. The red dashed lines highlight the significance threshold (|z| >2). Genes with negative z‐scores were associated with the benthic ecotype across lakes, and genes with positive thresholds were associated with pelagic ecotypes. Illustrations highlight differences in head shape between benthic and pelagic ecotypes. Core genes that were differentially expressed in at least two ecotype pairs and significantly associated with ecotype across lakes are highlighted in red, with gene IDs from the Arctic charr genome annotation. Chromosomes are highlighted by alternative colours, and unplaced scaffolds are located at the end

In this study, we investigated the patterns and contributions of differential gene expression, alternative splicing and genomic changes to rapid and parallel eco‐morphological divergence in these natural populations on a genome‐wide scale. Given that alternative splicing has been suggested to be independently evolving and less functionally constrained than gene expression, we hypothesized that (a) the genes and biological processes associated with alternative splicing would be different from those identified in differential expression analyses, (b) differentially spliced genes would be more central in co‐expression networks and pleiotropic, and (c) the associated genetic loci would differ between splicing and expression. As a consequence of reduced constraint, we hypothesized that (d) alternative splicing would be less and differently parallel across replicates compared to gene expression. We re‐analysed RNA‐sequencing (RNA‐seq) data generated from white muscle from three Arctic charr ecotype pairs across three independent lakes (Figure 1a, Table 1) for a previous analysis of genome‐wide gene expression (Jacobs et al., 2020). Here we performed more comprehensive and extensive analyses, integrating signals of selection and assessing patterns of alternative splicing using complementary approaches. This provides novel insights into the alternative roles of different molecular processes underlying rapid ecological and phenotypic divergence in an environmental context and fills an important knowledge gap in our understanding of the link between genotype, environment and adaptive phenotypes in natural populations.

**TABLE 1:** Overview table

Population	Latitude (N)/longitude (W)	Ecotypes	Evolutionary history	DE	DS (DIE/DEU)	Div. SNPs
Awe	56°20′/005°05′	Bn ‐ Pl	IM	209	1475 (85/1428)	471
Tay	56°30′/004°10′	Bn ‐ Pl	SC	692	197 (48/164)	357
Dughaill	57°28′/005°20′	Bn ‐ Pl	SC	167	148 (104/67)	396

Ecotypes: benthic (Bn) and pelagic (Pl); evolutionary histories: probably evolved under isolation‐with‐migration (IM), probably evolved under secondary contact (SC); DE, differentially expressed gene; DS, differentially spliced gene (based on differential intron excision (DIE) and differential exon usage (DEU); Div. SNPs, number of divergent SNPs showing signs of selection based on Rsb score.

## 2 MATERIAL AND METHODS

### 2.1 Data

RNA‐seq data were drawn from Jacobs et al. (2020) (NCBI BioProject: PRJNA551374). Briefly, adult Arctic charr had been sampled from Loch Awe, Loch Tay and Loch Dughaill in Scotland (Figure 1a, Table 1) during spawning time. High‐quality RNA was extracted from white muscle tissue and RNA‐seq libraries were prepared for 24 individuals (*n* = 4 per ecotype per lake) using the TruSeq Stranded mRNA Sample Preparation kit. Libraries were paired‐end sequenced to an average depth of 25–30 million reads per library.

### 2.2 Filtering and read mapping

Adapters and low‐quality reads were trimmed using trimmomatic version 0.36 and reads were aligned against the charr reference genome (ASM291031v2) (Christensen et al., 2018) with star version 2.5.2b using a two‐step mapping approach and duplicates were marked using the picard tool.

### 2.3 Gene expression analyses

Raw reads for each transcript were counted using htseq‐count, and subsequently filtered, normalized and analysed using deseq2. Transcripts with <20 reads across all samples were excluded. Principal component analysis (PCA) was performed based on *rlog*‐transformed read counts using pcamethods (R‐package) with the following settings: scaling = “none”, center = TRUE. First, we identified differentially expressed genes between benthic and pelagic ecotypes using a pairwise analysis by lake in deseq2. Second, we used a conditioned redundancy analysis (RDA) in vegan (R‐package) to identify genes with expression patterns associated with ecotype (binary), while correcting for lake effect. We selected genes with z‐transformed loadings above 2 or below −2 as associated with ecotype, corresponding to a *p*‐value of 0.05. Furthermore, we constructed signed gene co‐expression networks using wgcna (R‐package) based on *rlog*‐transformed read counts. Modules were defined using the dynamic treecut algorithm, with a minimum module size of 25 genes and a cut height of 0.992, and similar modules were merged using an eigengene distance threshold of 0.25. We assessed correlations of rank‐transformed module eigengene expression with lake, ecotype and sex using analyses of variance (ANOVAs). We used lake and sex as covariates when testing for correlations with ecotype. We further estimated the scaled intramodular connectivity (*k*_within_) for all genes within modules, excluding unassigned genes. We compared *k*_within_ between candidate genes (differentially spliced or expressed) and all other expressed genes using Wilcoxon rank sum tests.

### 2.4 Alternative splicing analyses

First, we used dexseq to analyse differential exon usage (DEU) between ecotypes while taking biological replicates into account. We used the modified htseq‐count script provided with dexseq to quantify exon‐specific read counts for each sample. Differential exon usage was estimated per lake using the following model structure: ~ sample + exon + ecotype:exon. A PCA based on *rlog*‐transformed exon expression counts was performed in pcamethods. Second, we used leafcutter (Li et al., 2018) to perform intron clustering and differential splicing analyses based on differential intron excision ratios (DIE) for each lake separately (minimum coverage of 10 reads per intron, minimum of four samples supporting an intron, minimum of two samples per ecotype supporting an intron). This method is independent of the genome annotation. Differential splicing of intron clusters was measured as the “change of per cent spliced in” (∆PSI). We used a combined data set for visualizing splicing patterns within and across lakes using a PCA based on intron cluster count ratios and sashimi‐plots using the leafviz shiny‐app that is distributed with leafcutter.

### 2.5 SNP calling, SNP effect prediction and signatures of selection

We called single nucleotide polymorphisms (SNPs) from the genome aligned RNA‐seq data using freebayes, after marking duplicates using picard, using a coverage threshold of three. We filtered the biallelic SNP data set using the *vcffilter* command in vcflib and vcftools, retaining biallelic SNPs with (a) phred quality score above 30, (b) genotype quality above 20 and (c) an allele‐depth balance between 0.25 and 0.75. We furthermore filtered for Hardy–Weinberg disequilibrium (*p*‐value threshold < .01) and only kept sites that were present in at least 90% of all individuals across populations. We annotated the retained SNPs and predicted their effect, particularly splice‐site disrupting variants, with snpeff. PCA was based on LD‐pruned SNPs in snprelate (R‐package). We identified SNPs putatively under selection by comparing patterns of extended haplotype homozygosity (Rsb score) between sympatric ecotypes using rehh (R‐package). We identified haplotypes under selection as those with absolute Rsb values above 4. Phasing and imputation were performed with fastphase.

### 2.6 Expression and splicing QTL mapping

We identified genetic variants putatively underlying variation in gene expression, and we used an expression quantitative trait locus (*cis*‐eQTL) approach to identify *cis*‐acting variants associated with expression. We used linear models with lake as a covariate implemented in matrixeqtl version 2.2 to associate SNPs with the expression of nearby genes (<1 Mb) and a false‐discovery rate below 0.05. Using the same approach, we also tested for *cis*‐regulatory splicing QTL (*cis*‐sQTL) based on association with intron excision ratios from leafcutter. Due to the limited sample size, we were not able to map *trans*‐acting sQTL or eQTL.

### 2.7 Functional gene ontology analysis

We used the gene ontology (GO) annotation provided with the published Arctic charr reference genome (Christensen et al., 2018) as the basis for analyses of biological processes and molecular functions. We used blast2go version 5.2.4 to perform overrepresentation analysis using Fisher's Exact tests and gene set enrichment analysis (GSEA). We clustered GO terms using the _REVIGO_ clustering algorithm to account for redundancy, and visualized similarities between clustered GO terms using multidimensional scaling (MDS) scaling plots based on the semantic similarities of GO terms, as well as interaction networks in cytoscape.

### 2.8 Statistical analysis of sharing across lakes

We performed hypergeometric tests using the phyper R‐function to calculate the probability that differentially regulated genes are shared more or less often across two lakes than expected by chance. To identify if more or fewer genes were shared than expected, we calculated a representation factor (RF), which compared the observed number shared genes to the expected number (e.g. [Number of DS × Number of DE]/all expressed genes). An RF >1 indicates that more genes than expected are shared, while an RF <1 indicates that fewer than expected are shared (Grantham & Brisson, 2018).

## 3 RESULTS

### 3.1 Divergence in gene expression between ecotypes

We investigated patterns of gene expression divergence between sympatric Arctic charr ecotypes within and across the three lakes (Figure 1a, Table 1). PCA based on gene expression data for 19,623 transcripts showed that the lake of origin term had the strongest effect on expression patterns (PC1 = 24.9%; linear model [LM] effect size for PC1: η^2^_Lake_ = 0.816, *p* < .001; Figure 1b), consistent with the independent divergence history of ecotype pairs since the last glacial maximum (Jacobs et al., 2020). Benthic and pelagic ecotypes mainly separated along PC2 (12.8%; LM: η^2^_Ecotype_ = 0.296, *p* = .013) (Figure 1b), indicating consistent patterns of gene expression divergence along a major axis of variation. Parallelism was further supported by the nonsignificance of the “ecotype × lake interaction” term for PC1 (LM: η^2^_Ecotype × Lake _= 0.064, *p* = .552) and PC2 (LM: η^2^_Ecotype × Lake _= 0.136, *p* = .269), as a significant interaction term would represent nonparallel differences in gene expression across lakes. Overall, these results are consistent with trajectory analyses performed by Jacobs et al. (2020).

We detected between 169 and 692 differentially expressed (DE) genes between sympatric ecotypes (Figure 1c, Table 1; Figure S1, Table S1), with ecotype pairs from two different lakes sharing more DE genes than expected by chance (Hypergeometric tests [HGT]; *p* < .001, RF between 2.7 and 5.0; RF >1 indicates more overlap than expected). Combined analyses of expression across all samples and lakes using an RDA identified 699 genes that showed ecotype‐associated expression, with ecotype explaining 6.8% of gene expression variation (ANOVA: *F*_(1,20)_ = 1.914, *p* = .007) (Figure 1d; Figure S1b). Of these 699 ecotype‐associated genes, 23 were also detected as differentially expressed in at least two of the three populations (Table S1).

### 3.2 Patterns of differential splicing within and across lakes

**FIGURE 2:**
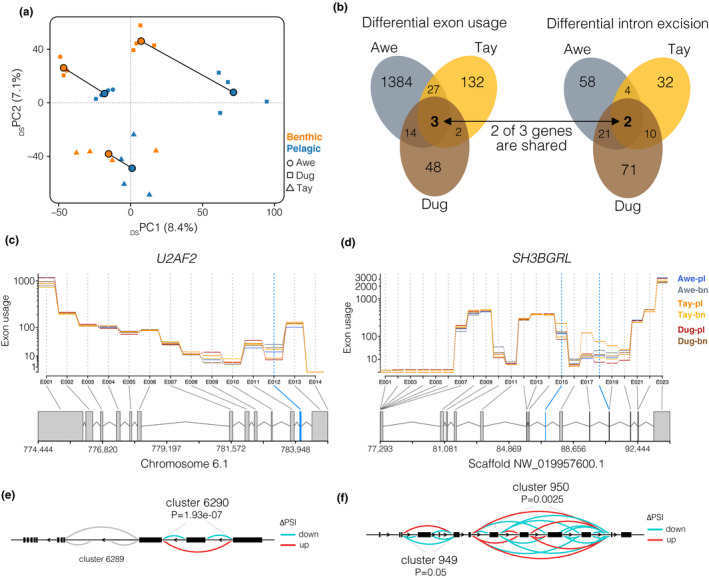
Shared patterns of differential splicing across lakes. (a) PCA based on intron excision ratios (*n* = 18,207 introns) for all individuals. See Figure 1 for explanation of the key. (b) Venn diagrams showing the amount of overlap of differentially spliced genes based on differential exon usage and differential intron excision between sympatric ecotypes across lakes. Two genes were detected using both approaches. (c, d) Gene models illustrating alternative splicing patterns for (c) *U2AF2* and (d) *SH3BGRL* based on exon usage. The expression of each exon corrected for overall gene expression (exon usage) is shown for each ecotype. Exons that are differentially spliced in at least two of the three ecotypes are highlighted in blue. (e,f) Sashimi graphs highlighting patterns of differential intron excision (DIE) across all ecotypes and lakes for intron clusters associated with (e) *U2AF2* and (f) *SH3BGRL*. The amount of DIE is measured as “change in the per cent spliced in (ΔPSI)”. The arcs represent splice junction connected exons, with the colour displaying if they are either up‐ or down‐regulated in the benthic compared to the pelagic ecotype

We investigated alternative splicing patterns within and across ecotype pairs using two independent approaches (see Methods). The lake of origin explained the largest proportion of variance in splicing based on intron excision ratios (*n* = 18,207 intron‐clusters; DIE) (PC1: LM: η^2^_lake _=0.878, *p* = 5.97e‐09; Figure 2a). The major axes of variation in splicing also strongly separated ecotypes (Figure 2a), with ecotype explaining a substantial proportion of the total variance along PC1 and PC2 (PC1 [8.4%]: η^2^_Ecotype_ = 0.729, *p* = 1.67e‐06; PC2 [7.1%]: η^2^_Ecotype_ = 0.593, *p* = 7.21e‐05). The nonparallel “lake × ecotype” interaction effect explained a significant proportion along PC1 (LM: η^2^_Ecotype × Lake _= 0.456, *p* = .0042), but not along PC2 (η^2^_Ecotype × Lake _= 0.272, *p* = .058), indicating some nonparallel variation in alternative splicing patterns across ecotype pairs. The PCA based on exon usage was similar to the gene expression PCA, with the major axis of variation in differential exon usage (DEU) explained by the lake of origin (DEU‐PC1 [23.3%]: LM: η^2^_lake_ = 0.802, *p* = 4.72e‐07; Figure S2a), and the ecotype and “lake × ecotype” interaction terms being nonsignificant (DEU‐PC1: η^2^_Ecotype _= 0.150, *p* = .0913; η^2^_Ecotype × Lake _= 0.072, *p* = .5125). Variation in exon usage along PC2 was significantly parallel across ecotype pairs, with the ecotype term explaining significant variation along PC2 (DEU‐PC1 [13.1%]: LM: η^2^_Ecotype _= 0.202, *p* = .0468; η^2^_lake _=0.016, *p* = .8670; η^2^_Ecotype × Lake _=0.141, *p* = .2537). Overall, these results suggest that major portions of variation in alternative splicing are parallel to across replicated ecotype pairs.

Analysing differential splicing between sympatric ecotypes, we found between 48 and 104 genes to be associated with differentially spliced intron clusters, and between 67 and 1428 genes to show differential exon usage (in one or several exons) (Figure 2b–f, Table 1), corresponding to about 1%–8% of all expressed genes. Of all differentially spliced genes, between 1.2% and 27.1% were detected in at least two of the three ecotype pairs (Figure 2b; Figures S2 and S3, Table S2; HGTs; all *p* < .05, RF between 33.9 and 51 for DIE; RF between 2.7 and 12.6 for DEU). Two genes, *U2AF2* and *SH3BGRL*, which encode for the splicing factor U2AF 65‐kDa subunit and the SH3 domain‐binding glutamic acid‐rich‐like protein, respectively, were differentially spliced in all three lakes based on DEU and DIE (Figure 2b–e; Figure S3a–d). Additionally, *FTH1*, which encodes the Ferritin Heavy Chain (Figure S3e), showed differential exon usage in all three lakes. The overall proportion of expressed genes that were differentially spliced was on average 2.82 ± 3.87% (mean ± *SE*) based on DEU and 0.40 ± 0.15% based on DIE.

Similarities in intron excision or exon usage patterns across lakes (e.g., conveyed in the PCA) do not necessarily mean that the same isoforms are being expressed in parallel ecotypes, but rather that exon or intron cluster counts are similar. However, by comparing splicing events across ecotype pairs we found evidence for parallelism on the isoform level. For example, differential intron excision, and therefore probably isoform usage, of *U2AF2* were identical between Tay and Awe (Figure S3a). Similarly, usage of exon 12 was consistently higher in all three benthic ecotypes (Figure S3c). Yet, in other cases splicing patterns of the same gene differed across lakes (Figure S3a,b). For example, in Dughaill a different intron cluster within *U2AF2* was differentially spliced (Figure S3a), the direction of differential intron excision differed between Tay and Dughaill in the second cluster of *SH3BGRL* (Figure S3b), and similarly the differences in exon usage of exon 18 in *SH3BGRL* were inconsistent between Awe and Tay (Figure S3c–e). In some cases, the number of differentially used exons also differed highly across lakes; for example, three exons showed significant DEU in Awe but only one in Tay and Dughaill, suggesting a putatively higher isoform diversity in Awe. Together, these results suggest that although differential splicing is parallel at the gene level, that isoform diversity is more variable across lakes, ranging from pronounced parallelism between some ecotype pairs to stark differences in isoform diversity between others.

### 3.3 Alternative splicing and differential gene expression are functionally nonredundant

**FIGURE 3:**
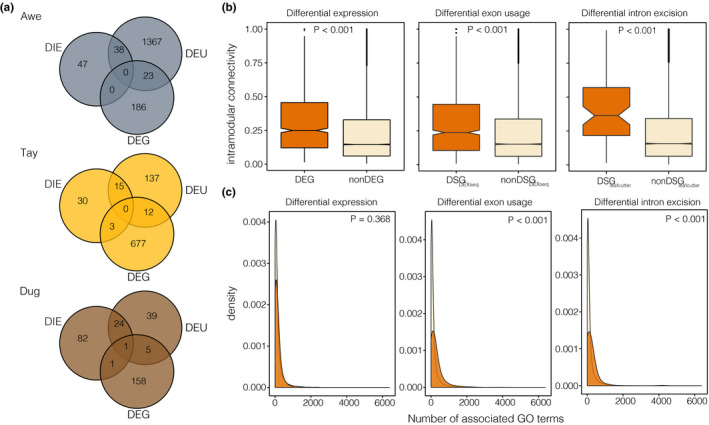
Sharing, connectivity and pleiotropy of differentially spliced and differentially expressed genes. (a) Venn diagrams showing the amount of overlap of differentially spliced (by analyses) and differentially expressed genes for each lake. (b) Boxplots (bar = median; notch = confidence interval around the median, box range = range between third and first quartile [interquartile range]; whiskers = extend to furthest point [highest or lowest] no further than 1.5 times the interquartile range; points = outliers) showing differences in intramodular connectivity between candidate and noncandidate genes for DEGs (*n* = 964 vs. *n* = 15,391), DEU (*n* = 926 vs. *n* = 16,355) and DIE (*n* = 103 vs. *n* = 16,355). (c) Differences in the number of associated gene ontology (GO) terms (biological processes) for candidate genes and noncandidate genes, based on DEGs and DSGs (DEU and DIE). DS genes were associated with more GO terms compared to the genomic background (noncandidate genes), suggesting higher pleiotropic effects

We found that differentially spliced (DS) genes (both DEU and DIE combined) were generally not differentially expressed, with only 4.9 ± 3.3% of DS genes being differentially expressed within ecotype pairs (1.6% in Awe, 8.2% in Dug to 4.8% in Tay; Figure 3a). While DS genes identified based on DIE were not differentially expressed (HGT: *p* > .05, RF between 0 and 1.77), DS genes based on DEU tended to be differentially expressed more often than expected by chance in all three ecotype pairs (HGT: *p* < .05, RF between 1.5 and 8.7). However, this overrepresentation could also stem from technical artefacts, namely that both dexseq (DEU) and deseq2 (DE) analyses rely on read count data of known annotated features (Anders et al., 2012). For example, differential expression of a gene or transcript can stem from the overexpression of only one exon (or isoform) rather than gene‐wide upregulation (Verta et al. 2020).

### 3.4 Increased connectivity and pleiotropy of candidate genes

To better understand the regulatory context and putative importance of DS and DE genes, we investigated gene co‐expression networks (Langfelder & Horvath, 2008) and pleiotropy based on GO annotations (McGirr & Martin, 2018). We found that both DE and DS genes showed higher degrees of connectivity than non‐DE (Wilcoxon rank sum test: *p* < .001) or non‐DS genes (Wilcoxon rank sum test: *p* < .001) in gene co‐expression networks (Figure 3b; Figures S4–S6; Supplementary results), consistent with more central positions of differentially regulated genes in regulatory expression networks.

DS genes were on average associated with more GO terms (biological processes) than non‐DS genes (Figure 3c), indicating a greater level of pleiotropy. DS genes were on average annotated with 265 (*SD *= 412) and 194 (*SD *= 369) GO terms for DEU (dexseq) and DIE (leafcutter), respectively, whereas non‐DS genes were annotated with an average of 142 (*SD *= 264) and 141 (*SD *=* *263) GO terms (Wilcoxon rank sum tests; DEU: *p* < .001; DIE: *p* < .001). We did not find any difference in the number of annotated GO terms between DE (mean = 141, *SD *= 245) and non‐DE genes (mean =148, *SD *= 289; Wilcoxon rank sum test: *p* = .368; Figure 3c). However, it is important to note that these inferences depend on the completeness of the GO annotation and assume that DE and DS genes are similarly well annotated.

Together, our results suggest that DS genes, and to a lesser degree DE genes, hold central functional roles in regulatory networks and are potentially more pleiotropic.

### 3.5 Different regulatory processes affect different biological pathways

**FIGURE 4:**
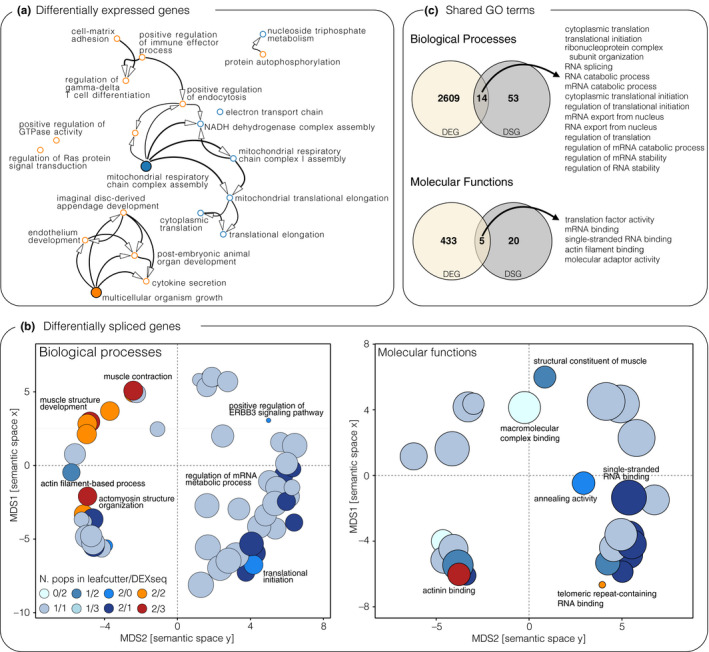
Functional pathways of divergence. (a) Gene ontology (GO) term interaction network for GO terms enriched for ecotype‐associated genes (RDA). Benthic ecotype associations are highlighted in orange, while pelagic ecotype associations are in blue. Central processes are highlighted by larger filled dots. (b) Multidimensional scaling (MDS) plots for shared GO terms (Biological processes; Molecular functions) enriched for differentially spliced genes (DIE and DEU). Clustering was performed based on semantic similarity of GO terms. Circles are coloured based on the number of populations they were enriched in and if they were detected based on DIE (leafcutter), DEU (dexseq) or both. The most representative and highly shared GO terms are named. The size of the circles corresponds to the number of genes associated with a GO term. (c) Venn diagrams showing the overlap between GO terms (Biological processes, Molecular functions) associated with differentially spliced (DS) and differentially expressed (DE) genes. The names of overlapping processes and functions are listed

We compared functional annotations (GO terms) of genes with ecotype‐associated gene expression (RDA analyses) and DS genes, to identify putative functional downstream differences. GO terms that were significantly enriched for ecotype‐associated DE genes (RDA analyses) in a GSEA were related to metabolic processes (e.g., oxidative phosphorylation) and translational activity (e.g., cytoplasmic translation) in pelagic ecotypes, and cell growth and differentiation (e.g., lymphocyte differentiation), immune response (e.g., interleukin‐1 secretion) and signal transduction (e.g., positive regulation of endocytosis) in benthic ecotypes (Figure 4a; Table S3). Similar processes and functions were identified using a GO term overrepresentation analysis of all significant ecotype‐associated DE genes, although none of the GO terms was significantly overrepresented after correcting for multiple testing (Table S4). In contrast, overrepresentation analysis of DS genes revealed an enrichment of biological processes and molecular functions related to muscle development and functioning (Figure 4b; Table S5), such as myofibril assembly, actinin binding and muscle structure development. Other processes enriched for DS genes included RNA‐binding processes, mRNA splicing and translational regulation (Figure 4b; Tables S3–S5). GO terms that were enriched for DE and DS genes were mostly involved in gene regulation, such as translation, transcription and RNA catabolism (Figure 4c; Figure S7). The proportions of overlap were relatively low, with an average of 13.0% (range from 0% to 25.8%) of DS‐enriched GO terms overlapping DE‐enriched GO terms (Figure S7). Overall, we conclude that DS and DE genes in white muscle are probably involved in different biological processes and have different molecular functions.

### 3.6 Genetic variation underlying gene regulation

**FIGURE 5:**
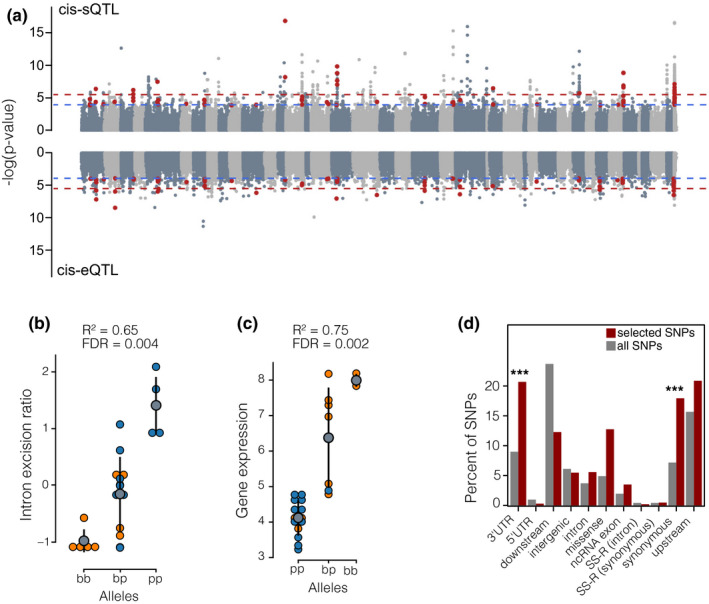
Genetic variation underlying regulatory variation. (a) Manhattan plots showing the association of SNPs with variation in alternative splicing (intron excision ratios) across ecotypes and lakes (top) and with variation in gene expression (bottom). SNPs that are highlighted in red were detected in both analyses (*n* = 117). Chromosomes are highlighted by alternative colours, and unplaced scaffolds are located at the end. The blue dashed line indicates a false‐discovery rate (FDR) of 5% and the red dashed line an FDR of 1%. (b) *cis*‐sQTL associated with the intron excision of an intron cluster located in *SRSF7*. The *y*‐axis shows the intron excision ratio for the intron cluster by genotype (p = pelagic allele, b = benthic allele) across individuals (points) and ecotypes (colour; orange = benthic, blue = pelagic). The grey dot and ranges show the mean intron excision ratio by genotype and the standard deviation. (c) *cis*‐eQTL associated with the normalized gene expression of *EGFR*. The plot shows how the expression of *EGFR* differs with genotype (p = pelagic allele, b = benthic allele) across individuals (points) and ecotypes (colour; orange = benthic, blue = pelagic). The grey dot and ranges show the mean expression per genotype and the standard deviation. Both *SRSF7* and *EGFR* are also differentially spliced or expressed in at least two ecotype pairs. (d) Predicted effects and locations of SNPs under selection. A larger proportion of SNPs under selection (in at least one lake) are located in 3′‐UTRs or are synonymous compared to proportions in the full SNP data sets (*p* < .001)

Lastly, we mapped the genetic basis of gene regulation and identified genetic signatures of selection to assess their impact on regulatory evolution. We identified 101,487 high‐confidence SNPs (Figure S8), of which 2404 (located in 2106 genes) were predicted to have effects on splicing using snpeff. Genes containing high‐impact splice‐site variants, SNPs at intron–exon boundaries that can lead to strong changes in splicing, were more likely to show differential intron excision (5.2% of all DS‐DIE genes; HGT: *p* = 8.54e‐15). We further identified 1,919 *cis*‐sQTL associated with variation in intron excision ratios of 626 intron clusters across all the three lake populations (false‐discovery rate [FDR] < .05, Figure 5a,b). Of these, five *cis*‐sQTL were predicted to be high‐impact splice variants, and 40 intron clusters associated with *cis*‐sQTL were also differentially spliced (HGT: *p* = .00013, RF = 22; expected intron clusters = 21; Table S6).

Furthermore, we identified 1,562 *cis*‐eQTL associated with the expression variation of 734 genes (FDR < .05, Figure 5a), with between 6.7 and 10.1% of DE genes being associated with *cis*‐eQTL (Figure 5c; Table S7). We found 117 *cis*‐sQTL and *cis*‐eQTL to be shared (HGT: *p* = 6.14e^−08^, RF = 70.4; expected number of shared QTL = 67), suggesting some genomic regions regulate both splicing and expression in Arctic charr. Yet, the majority of *cis*‐regulatory regions (96.64%) only affected one of the two regulatory processes. Overall, this suggests that differential splicing and expression are both at least in part genetically regulated, largely through independent regulatory variants.

We tested if differentially regulated genes were associated with genetic signatures of selection (Table S8). Neither DE nor DS genes were significantly associated with genetic signatures of selection (HGT: all *p* > .05). Only two *cis*‐sQTL and two *cis*‐eQTL showed signs of selection in at least one lake. However, SNPs putatively under selection in at least one population were more often located in the 3′ untranslated region (3′‐UTR) compared to the full SNP data set (20.7% of selected SNPs vs. 8.98% of all SNPs; χ^2^ = 4.53, *p* = .033) and were significantly more often synonymous mutations (17.94% of selected SNPs vs. 7.17% of all SNPs; χ^2^ = 4.35, *p* = .037) (Figure 5d). Thus, putatively selected SNPs might underlie gene expression changes between ecotypes through changes of *cis*‐regulatory elements in the 3′‐UTR rather than structural protein changes.

## 4 DISCUSSION

Post‐transcriptional processes are widely understudied in evolutionary and ecological genomics of natural populations, leading to a knowledge gap in our understanding of their role as a molecular mechanism of adaptation. Our transcriptomic analysis from ecologically relevant tissue (swimming muscle) from three independent and replicated benthic–pelagic Arctic charr ecotype pairs revealed novel insights into distinct regulatory patterns for alternative splicing and gene expression. We found evidence suggesting that alternative splicing and differential expression, which show parallel and nonparallel aspects across ecotype pairs, play contrasting but complementary functional roles by affecting different genes and pathways. These processes are probably regulated through alternative genetic changes and plastic responses, as evidenced by our integration of expression and splicing QTL mapping with selection analyses. Alternative splicing potentially facilitated the rapid adaptive evolution of Arctic charr due to the central position in regulatory networks and putatively increased pleiotropy of differentially spliced genes. Overall, this study provides novel insights into the molecular processes underlying adaptive parallel divergence and suggests alternative roles for complementary regulatory processes in rapid and parallel adaptive diversification.

### 4.1 Alternative splicing and gene expression have contrasting phenotypic effects

Contrary to other studies of adaptation or acclimation in teleost fishes (Healy & Schulte, 2019; Singh et al., 2017), we found that DS genes were generally not differentially expressed between ecotypes (Figure 3a). This lack of overlap agrees with our initial hypotheses that splicing and expression affect different biological pathways and downstream phenotypes, presumably due to being independently regulated (Li et al., 2016). Indeed, DS and DE genes were enriched for different biological processes and molecular functions (Figure 4; Figure S7). DS genes were mostly involved in processes related to muscle development and function through the alternative splicing of genes with molecular functions involved in actinin binding (Figure 4b). Functional differences in muscle formation and functioning are consistent with differences in swimming performance and activity between benthic and pelagic ecotypes that require adaptive changes in muscle composition and arrangement (Altringham & Ellerby, 1999; Klemetsen, 2002). In contrast, DE genes were mostly enriched for metabolic and developmental processes (e.g., mitochondrial respiratory chain complex, post‐embryonic organ development) (Figure 4a), consistent with putative differences in activity and metabolic requirements between ecotypes with different foraging and swimming habits (Dalziel et al., 2018; Evans & Bernatchez, 2012; Klemetsen, 2002). Metabolic processes in particular have been observed to be differentially regulated based on expression in other cases of eco‐morphological diversification in teleosts (Filteau et al., 2013; McGirr & Martin, 2018) and were found to be under selection in salmonid fishes (Schneider et al., 2019). Thus, alternative splicing analyses in other adaptive radiations might reveal genes and pathways that have previously not been not implied in their eco‐morphological diversification.

Alternative splicing in vertebrates has been suggested to diverge more rapidly than gene expression (Barbosa‐Morais et al., 2012; Merkin et al., 2012), indicating that it might play a key role at early stages of divergence and ecological speciation. However, these previous studies compared the evolution of expression and splicing over the span of millions of years. In contrast on the much more recent timescales examined in our study, we did not detect a consistently higher number of either DS or DE genes across all three Arctic charr ecotype pairs (Figure 3a), suggesting that splicing and expression might evolve at similar or inconsistent rates. If splicing evolves faster than gene expression, we speculate that the divergence in muscle development and function in Arctic charr, which is associated with DS genes, might have preceded the metabolic divergences associated with DE genes. Comparative studies across replicated ecotype pairs with different divergence times are needed to better understand the role of splicing in rapid eco‐morphological divergence on postglacial timescales.

The alternative splicing of transcripts can either lead to new functional proteins or to the regulation of transcript abundance through splicing‐related nonsense‐mediated decay (NMD) (Stamm et al., 2005). The role of NMD in regulating transcript abundance between ecotypes is supported by the differential splicing and regulation of *SRSF7* (Figure 5b), a splicing factor that has been linked to regulating the NMD pathway (Königs et al., 2020). In general, genes involved in the transcription and splicing machinery were both differentially expressed and spliced (Figure 4c), suggesting that gene regulatory processes are highly divergent between ecotypes. This includes *U2AF2* on linkage group (LG) 6.1 (Figure 2c,e), which is differentially spliced in all three ecotype pairs and codes for a splicing factor important for pre‐mRNA processing. Interestingly, we observed that a paralogue of *U2AF2* on LG 33 was differentially expressed between ecotypes. This suggests that paralogues, which potentially originated through the whole‐genome duplication in salmonids (Lien et al., 2016), might be regulated through different molecular processes, thus increasing functional diversity while minimizing constraints (Bush et al., 2017; Iñiguez & Hernández, 2017). However, more analyses are needed to test this hypothesis (Iñiguez & Hernández, 2017) and assess the role of the whole‐genome duplication in salmonids on the regulatory divergence between recently diverged ecotypes. While we cannot discern the exact molecular and phenotypic impact of splicing and expression events without detailed molecular studies of translation, protein abundance and transcript decay for candidate genes (Mallarino et al., 2017), our findings are consistent with our hypothesis that alternative splicing and gene expression play contrasting functional roles in the divergence of Arctic charr ecotypes.

### 4.2 Parallel divergence in alternative splicing across replicated ecotype pairs

While it is known that gene expression patterns are often highly parallel across ecotype pairs, even across lineages (Filteau et al., 2013; Jacobs et al., 2020; Manousaki et al., 2013; Rougeux et al., 2019), less is known about patterns of alternative splicing in ecological context. For example, parallel splicing has been described across cichlid species occupying parallel trophic niches (Singh et al., 2017), suggesting that alternative splicing might play a role in the evolution and development of replicated adaptive phenotypes. We found parallel patterns of alternative splicing across the three replicated ecotype pairs (Figure 2a), with dozens of genes being differentially spliced in two or all three ecotype pairs (Figure 2b). Contrary to our hypothesis, the similar parallelism in splicing and expression on the gene level that we found suggests the presence of regulatory constraints or low redundancy on splicing, for example due to limited standing regulatory variation or strong selection on adaptive genes/pathways across all three populations (Rougeux et al., 2019; Yeaman, 2015).

Despite the fact that some genes showed evidence for differential splicing across multiple ecotype pairs, we found that the exact splicing events were in some cases more variable across lakes (Figure 2c–f). While *U2AF2*, *SH3BGRL* and *FTH1* were detected to be differentially spliced in all three ecotype pairs, the exact differentially expressed exon (dexseq) or excised intron cluster (leafcutter) differed in some cases between lakes (Figure S3). The same intron clusters in *U2AF2* were differentially spliced in Awe and Tay (Figure S3a), but in Dughaill an overlapping but distinct intron cluster was differentially spliced (Figure S3a), probably leading to the formation of a different isoform. Such isoform differences can probably lead to differences in the function and/or fate of transcripts, and can therefore have drastically different downstream effects (Eksi et al., 2013; Keren et al., 2010; Mallarino et al., 2017; Verta et al., 2020). Such differences and similarities in splicing across lakes further suggest that the underlying genetic changes and/or splicing mechanisms might be variable across lakes, due to either different environmental impacts that alter splice‐site choice or differences in the regulatory genetic architectures (Keren et al., 2010). The differential splicing of the same genes, despite the usage of different isoforms, indicates a potential functional role of these genes in the ecological divergence of Arctic charr, although the functional role of these isoforms might differ. To understand fine‐scale differences in isoform diversity, isoform‐level analysis, based on either reconstructed isoforms from short‐read RNA‐seq data or full‐transcript long‐read sequencing, will be needed. This would ideally be coupled with proteomic and functional analyses of isoforms to better understand the downstream impacts of such splicing differences.

### 4.3 Central regulatory roles and pleiotropy of alternatively spliced genes

The effect of differential regulation on phenotypic divergence has been suggested to be stronger for genes that are more central in regulatory networks (“hub genes”) and show a higher degree of pleiotropy (Batada et al., 2006; Filteau et al., 2013), as simple changes to such central genes can have rapid and substantial phenotypic effects (Koubkova‐Yu et al., 2018). On the other hand, it has been suggested that DS genes are less likely to be hub genes due to constraints of strong co‐expression correlations and potentially increased pleiotropy (Iñiguez & Hernández, 2017). However, we found that DS and DE genes were highly central in regulatory networks (Figure 3b), more so than non‐DE and non‐DS genes, which agrees with our hypothesis and suggests that the differential regulation of these genes potentially has strong functional impacts on the divergence of Arctic charr.

Constraints associated with the high complexity of altering pleiotropic or hub genes has led to the suggestion that they are less important for rapid adaptive evolution (Mäkinen et al., 2016; Papakostas et al., 2014). For example, lower pleiotropy of DE genes has been found in other cases of rapid adaptation, such as in pupfishes or European grayling (McGirr & Martin, 2018; Papakostas et al., 2014). In contrast to these previous studies and in agreement with our initial hypothesis, we found that DS genes were highly central and showed high levels of pleiotropy (Figure 3b,c). This supports the idea that splicing is less constrained on the functional level and suggests that alternative splicing might provide a mechanism through which the function or expression of pleiotropic hub genes can be altered or diversified without derailing vital expression patterns or coding sequence (Bush et al., 2017). This is also in line with previous findings that the differential splicing of large‐effect genes was associated with stark phenotypic evolution in other species (Howes et al., 2017; Mallarino et al., 2017; Verta et al., 2020). However, the opposite has been argued in other studies (Iñiguez & Hernández, 2017). This discrepancy highlights the need for more large‐scale comparative and functional studies of alternative splicing in adaptation and in ecological context to better understand its functional role and the underlying regulatory mechanisms.

### 4.4 Genetic regulation of alternative splicing and gene expression

Phenotypic divergence between Arctic charr ecotypes has been suggested to be affected by both heritable genetic variation and phenotypic plasticity (Adams & Huntingford, 2002a,2002b,^2004^; Klemetsen, 2002). By mapping *cis*‐regulatory expression and splicing QTL, we show that differential expression and differential splicing are at least partially genetically determined, with between 6.7% and 10.1% of DE genes and 16.5% of differentially spliced intron clusters being associated with *cis*‐regulatory variation. The support for our hypothesis about genetic regulation was equivocal, as a large proportion of QTL loci differed between DE and DS genes but the overlap between them was more than expected by chance alone.

*Cis*‐regulatory divergence has been shown to play an important role in rapid and parallel adaptive divergence in other species (Prud’homme et al., 2007; Verta & Jones, 2019; Wittkopp et al., 2008). Consistent with previous findings for *cis*‐regulatory expression QTL in Arctic charr and other postglacial fishes (Jacobs et al., 2020; Rougeux et al., 2019; Verta & Jones, 2019), *cis*‐regulatory QTL regulated the differential splicing of genes across replicated ecotypes in multiple cases (Figure 5b,c), suggesting an importance of shared adaptive regulatory variation in parallel evolution. However, due to our limited sample size, we were only able to map the strongest effect loci and could not detect *trans*‐QTL, meaning that our calculation of the genetic regulation of differential regulation is probably an underestimate. Studies of splicing regulation related to domestication in sunflowers suggest that *trans*‐sQTL play an important role in the evolution and regulation of splicing in some systems (Smith et al., 2018), and *trans*‐regulatory divergence, for example, has been suggested to be more important for intraspecific regulatory evolution compared to *cis*‐regulatory divergence (McGirr & Martin, 2020), although these patterns are not always consistent (Verta & Jones, 2019). In general, the vast majority (96.64%) of *cis*‐regulatory QTL either regulated splicing or expression but not both, consistent with findings in humans (Li et al., 2016). This might be due to the fact that *cis*‐eQTL are mostly located close to transcription start sites, while *cis*‐sQTL are often located within gene bodies, although both can simultaneously affect the same gene and are not exclusive (Lee & Rio, 2015; Li et al., 2016). This difference in regulatory architecture potentially plays a small role in explaining why different gene sets were differentially spliced and expressed (Figure 3a). Overall, it will be important to study the regulatory architecture underlying differential splicing in the context of ecological adaptation under controlled environmental conditions to understand the impact of the environment on the observed differences in splicing and expression.

Similar to many other studies, we did not find any evidence for signatures of selection within differentially regulated genes (Hodgins et al., 2016; Renaut et al., 2012). Surprisingly, in addition neither *cis*‐sQTL nor *cis*‐eQTL (Figure 5a) were significantly associated with signatures of selection. However, we found that sites putatively under selection were probably located in regulatory regions (e.g., 3′UTR), suggesting that they affect gene expression. One possible explanation is that sites under selection are associated with expression and or splicing divergence in tissues other than muscle, as expression and splicing patterns and regulation often drastically differ across tissues (Saha et al., 2017). Overall, our results indicate that both differential expression and splicing are in part genetically regulated and that these *cis*‐regulatory loci are in part parallel across lakes, which is in agreement with our earlier findings (Jacobs et al., 2020).

### 4.5 Limitations and future work

Our study provides novel insights into the role of post‐transcriptional processes in facilitating the rapid and parallel postglacial adaptive divergence in ecological context. Our use of complementary approaches, combining annotation‐based exon counts in dexseq and annotation‐free inferences of intron excision in leafcutter, overcomes the challenges and limitations of alternative splicing analyses based on short‐read data, such as the incomplete annotation or reconstruction of isoforms, in natural populations of a nonmodel organism. Thus, several lines of evidence support our inferences. While the application of novel analytical approaches to existing data can provide new fundamental insights (Jacobs et al., 2018), the rise of long‐read and direct RNA‐seq will facilitate a better understanding of these processes in natural populations.

One important point to be noted is that isoforms can have different or novel functions that are putatively not annotated, and functional annotations of nonmodel species are potentially incomplete (Bush et al., 2017; Eksi et al., 2013). Thus, the GO term‐based functional inference is limited and probably conservative. Without functional assays of translation and protein function, which are beyond the scope of this study, we cannot infer the potential of novel or alternative downstream functional roles for various isoforms (Mallarino et al., 2017; Stamm et al., 2005). However, the fact that DS and DE genes were largely associated with different processes and functions suggests strongly that these alternative processes mostly play contrasting roles. Overall, to better understand the role of alternative splicing in rapid ecological adaptation, future work should focus on (a) studying alternative splicing under different adaptive scenarios and timescales, (b) identifying the regulatory basis in laboratory and wild settings, (c) using novel long‐read approaches to quantify and compare isoform diversity under different evolutionary, genomic and environmental backgrounds, and (d) using functional assays to better understand the downstream impacts of alternative splicing.

## 5 CONCLUSIONS

Here, we provide important and novel insights into the role of alternative splicing in parallel evolution of natural populations. We suggest that alternative splicing and gene expression in freshwater fish white muscle affect different axes of phenotypic variation; splicing mostly causes structural and functional changes in the muscle and differential expression mostly leading to differences in energy metabolism. Together, we suggest these complementary regulatory processes facilitate the rapid adaptive divergence between Arctic charr ecotypes in foraging and swimming performance. We further show that differentially spliced genes probably play central regulatory roles. This study provides a putative mechanistic framework by which the concerted modification of alternative regulatory processes might facilitate the rapid and parallel adaptive eco‐morphological divergence in a postglacial fish.

## AUTHOR CONTRIBUTIONS

A. J. and K. R. E conceived the project. A. J. conducted all analyses and wrote the initial manuscript. Both authors contributed to the writing of the final manuscript.

## Supporting information

Supplementary Material

## Data Availability

The RNA‐seq data used for this project are available on NCBI under the BioProject PRJNA551374 with the following Run accessions: SRR9599659–SRR9599668, SRR9599680–SRR9599689 and SRR9599697–SRR9599700. Transcript counts for expression analysis, exon count tables for the exon usage analyses in dexseq, intron count tables and differential intron excision results from the intron excision analysis in leafcutter, and the VCF file with SNPs called from RNA‐seq data are deposited in the University of Glasgow Enlighten repository (http://dx.doi.org/10.5525/gla.researchdata.1097).
